# Developing a Model to Predict Hospital Encounters for Asthma in Asthmatic Patients: Secondary Analysis

**DOI:** 10.2196/16080

**Published:** 2020-01-21

**Authors:** Gang Luo, Shan He, Bryan L Stone, Flory L Nkoy, Michael D Johnson

**Affiliations:** 1 Department of Biomedical Informatics and Medical Education University of Washington Seattle, WA United States; 2 Care Transformation Intermountain Healthcare Salt Lake City, UT United States; 3 Department of Pediatrics University of Utah Salt Lake City, UT United States

## Abstract

**Background:**

As a major chronic disease, asthma causes many emergency department (ED) visits and hospitalizations each year. Predictive modeling is a key technology to prospectively identify high-risk asthmatic patients and enroll them in care management for preventive care to reduce future hospital encounters, including inpatient stays and ED visits. However, existing models for predicting hospital encounters in asthmatic patients are inaccurate. Usually, they miss over half of the patients who will incur future hospital encounters and incorrectly classify many others who will not. This makes it difficult to match the limited resources of care management to the patients who will incur future hospital encounters, increasing health care costs and degrading patient outcomes.

**Objective:**

The goal of this study was to develop a more accurate model for predicting hospital encounters in asthmatic patients.

**Methods:**

Secondary analysis of 334,564 data instances from Intermountain Healthcare from 2005 to 2018 was conducted to build a machine learning classification model to predict the hospital encounters for asthma in the following year in asthmatic patients. The patient cohort included all asthmatic patients who resided in Utah or Idaho and visited Intermountain Healthcare facilities during 2005 to 2018. A total of 235 candidate features were considered for model building.

**Results:**

The model achieved an area under the receiver operating characteristic curve of 0.859 (95% CI 0.846-0.871). When the cutoff threshold for conducting binary classification was set at the top 10.00% (1926/19,256) of asthmatic patients with the highest predicted risk, the model reached an accuracy of 90.31% (17,391/19,256; 95% CI 89.86-90.70), a sensitivity of 53.7% (436/812; 95% CI 50.12-57.18), and a specificity of 91.93% (16,955/18,444; 95% CI 91.54-92.31). To steer future research on this topic, we pinpointed several potential improvements to our model.

**Conclusions:**

Our model improves the state of the art for predicting hospital encounters for asthma in asthmatic patients. After further refinement, the model could be integrated into a decision support tool to guide asthma care management allocation.

**International Registered Report Identifier (IRRID):**

RR2-10.2196/resprot.5039

## Introduction

### Background

In the United States, asthma affects 8.4% of the population and leads to 2.1 million emergency department (ED) visits, 479,300 hospitalizations, 3388 deaths, and US $50.3 billion in cost annually [1,2]. Reducing hospital encounters, including inpatient stays and ED visits, is highly desired for asthmatic patients. For this purpose, using prognostic predictive models to prospectively identify high-risk asthmatic patients and enroll them in care management for tailored preventive care is deemed state of the art and has been adopted by health plans in 9 of 12 metropolitan communities [3]. Once enrolled, care managers make regular phone calls to help patients book appointments and schedule health and related services. If done properly, this can cut the patients’ future hospital encounters by up to 40% [4-7].

Unfortunately, the current high-risk patient identification methods have major gaps, leading to suboptimal outcomes. Care management typically enrolls only 1% to 3% of patients because of capacity constraints [8]. The existing models for predicting hospital encounters in asthmatic patients are inaccurate, which is reflected by their area under the receiver operating characteristic curve (AUC) ≤0.81 [9-22]. When used for care management, these models miss over half of the patients who will incur future hospital encounters and incorrectly classify many other patients as patients who will incur future hospital encounters. This makes it difficult to align care management enrollment with the patients who will actually incur future hospital encounters, increasing health care costs and impairing patient outcomes. If we could find 5% more asthmatic patients who would incur future hospital encounters and enroll them in care management, we could improve outcomes and avoid up to 9850 inpatient stays and 36,000 ED visits each year [1,4-7].

### Objectives

The goal of this study was to develop a more accurate model for predicting hospital encounters for asthma in asthmatic patients. The dependent variable is categorical with 2 possible values: whether future hospital encounter for asthma will occur or not. Accordingly, our model employs clinical and administrative data to perform binary classification, with the intention to better guide care management allocation and improve outcomes for asthmatic patients. A description of the development and evaluation of our model follows.

## Methods

### Study Design and Ethics Approval

In this study, we conducted secondary analysis of retrospective data. The study was reviewed and approved by the institutional review boards of Intermountain Healthcare, University of Utah, and University of Washington Medicine.

### Patient Population

Our patient cohort was based on the patients who visited Intermountain Healthcare facilities during 2005 to 2018. Intermountain Healthcare is the largest health care system in the Intermountain region (Utah and southeastern Idaho), with 185 clinics and 22 hospitals providing care for approximately 60% of the residents in that region. The patient cohort included asthmatic patients identified as residents of Utah or Idaho, with or without a specific home address. A patient was defined as having asthma in a given year if the patient had at least one diagnosis code of asthma (International Classification of Diseases, Ninth Revision [ICD-9]: 493.0x, 493.1x, 493.8x, and 493.9x; International Classification of Diseases, Tenth Revision [ICD-10]: J45.x) in that year in the encounter billing database [11,23,24]. Patients who died during that year were excluded. There were no other exclusions.

### Prediction Target (Dependent Variable)

In the rest of this paper, we use hospital encounter for asthma to refer to inpatient stay or ED visit at Intermountain Healthcare with a principal diagnosis of asthma (ICD-9: 493.0x, 493.1x, 493.8x, and 493.9x; ICD-10: J45.x). For each patient meeting criteria for asthma in a given year, we looked at any hospital encounter for asthma in the following year as outcome. In our modeling, we used each asthmatic patient’s data by the end of each year to predict the patient’s outcome in the following year.

### Dataset

The Intermountain Healthcare enterprise data warehouse provided a structured, clinical, and administrative dataset, including all visits of the patient cohort at Intermountain Healthcare facilities during 2005 to 2018.

### Features (Independent Variables)

Following the approach outlined in our study design papers [25,26], we considered 235 candidate features derived from the structured attributes in our dataset. These features came from 4 sources: the >100 potential risk factors for asthma exacerbations reported in the literature [9,22,27-34]; features used in the existing models for predicting asthma exacerbations [9-22]; factors impacting patients’ general health status mentioned in the literature [31,35,36]; and features suggested by the clinical experts in our team—MDJ, BLS, and FLN. As the characteristics of the patient, the care provider, and the treating facility impact the patient’s outcome, we used patient features as well as provider and facility features [25,26].

The 235 candidate features are listed in the first table in Multimedia Appendix 1 [37-39], where each reference to the number of a specific type of items, such as medications, counts multiplicity, unless the word *distinct* appears. A major visit for asthma is defined as an outpatient visit with a primary diagnosis of asthma, an ED visit with an asthma diagnosis code, or an inpatient stay with an asthma diagnosis code. An outpatient visit with asthma as a secondary diagnosis is defined as a minor visit for asthma. Intuitively, all else being equal and compared with a patient with only minor visits for asthma, a patient with 1 or more major visits for asthma is more likely to incur future hospital encounters for asthma.

Each input data instance for the predictive model includes the 235 candidate features, targets the unique combination of an asthmatic patient and a year (index year), and is used to predict the patient’s outcome in the following year. For that combination of patient and year, the patient’s age, current primary care provider (PCP), and home address were determined based on the data available on the last day of the index year. The features of premature birth, bronchiolitis, duration of asthma, duration of chronic obstructive pulmonary disease, whether the patient had any drug or material allergy, whether the patient had any environmental allergy, whether the patient had any food allergy, and the number of allergies of the patient were derived from the historical data from 2005 to the index year. Furthermore, 1 feature was derived from the historical data in both the index year and the year before. This feature is as follows: the proportion who incurred hospital encounters for asthma in the index year out of all asthmatic patients of the patient’s current PCP in the year before. The remaining 226 features were derived from the historical data in the index year.

### Data Analysis

#### Data Preparation

For every numerical feature, we checked the data distribution, adopted the following lower and upper bounds to spot invalid values, and replaced them with null values. Using the lower and upper bounds from the Guinness World Records [40], all body mass indexes <7.5 or >204, all weights <0.26 kg or >635 kg, and all heights <0.24 m or >2.72 m were deemed physiologically impossible and invalid. Using the lower and upper bounds provided by our team’s clinical expert MDJ, all peripheral capillary oxygen saturation values >100%, all temperatures <80°F or >110°F, all systolic blood pressure values ≤0 mm Hg or >300 mm Hg, all diastolic blood pressure values ≤0 mm Hg or >300 mm Hg, all heart rates <30 beats per minute or >300 beats per minute, and all respiratory rates >120 breaths per minute were deemed physiologically impossible and invalid.

To put all the numerical features on the same scale, we standardized every numerical feature by first subtracting its mean and then dividing by its standard deviation. As outcomes were from the following year, our dataset provided 13 years of effective data (2005-2017) over a total of 14 years (2005-2018). To reflect the model’s use in practice, data from 2005 to 2016 were used to train predictive models. Data from 2017 were used to assess the model’s performance.

#### Performance Metrics

As shown in the formulas below and Table 1, we applied 6 standard metrics to gauge the model’s performance: AUC, accuracy, sensitivity, specificity, positive predictive value (PPV), and negative predictive value (NPV).

The following formulas were used to calculate the standard metrics to gauge the model’s performance:

- Accuracy=(TP+TN)/(TP+TN+FP+FN)
- Sensitivity=TP/(TP+FN)
- Specificity=TN/(TN+FP)
- PPV=TP/(TP+FP)
- NPV=TN/(TN+FN)

Here, TP is true positive, TN is true negative, FP is false positive, and FN is false negative. For example, FN is the number of patients who will incur future hospital encounters for asthma and whom the model incorrectly projects to incur no future hospital encounter for asthma. Sensitivity shows the proportion of patients who will incur future hospital encounters for asthma found by the model. Specificity shows the proportion of patients who will incur no future hospital encounter for asthma found by the model.

For the 6 performance metrics, we obtained their 95% CIs via 1000-fold bootstrap analysis [41]. We calculated our final model’s performance metrics on every bootstrap sample of the 2017 data. For each performance metric, we got 1000 values, the 2.5th and 97.5th percentiles of which gave its 95% CI. We drew the receiver operating characteristic curve to exhibit the sensitivity-specificity trade-off.

**Table 1:** The confusion matrix.

Class	Future hospital encounters for asthma	No future hospital encounter for asthma
Predicted future hospital encounters for asthma	True positive	False positive
Predicted no future hospital encounter for asthma	False negative	True negative

#### Classification Algorithms

We used Waikato Environment for Knowledge Analysis (Weka), version 3.9 [42], to construct machine learning classification models. Weka is a widely used, open-source machine learning and data mining package. It incorporates many standard machine learning algorithms and feature selection techniques. We considered the 39 native machine learning classification algorithms in Weka listed in Multimedia Appendix 1 as well as the extreme gradient boosting (XGBoost) classification algorithm [43] implemented in the XGBoost4J package [44]. An XGBoost model is an ensemble of decision trees formed in a stagewise manner. As a scalable and efficient implementation of gradient boosting, XGBoost adopts a more regularized model formulation to help avoid overfitting and improve classification accuracy. We used our previously developed automatic model selection method [45] and the 2005 to 2016 training data to automate the selection of the machine learning classification algorithm, feature selection technique, data balancing method for handling imbalanced data, and hyperparameter values among all the suitable ones. Our automatic model selection method [45] adopts the response surface methodology to automatically check many combinations of classification algorithm, feature selection technique, data balancing method, and hyperparameter values and conducts cross-validation to choose the final combination to maximize the AUC. AUC has no reliance on the cutoff threshold used for deciding between the projected future hospital encounters for asthma and the projected no future hospital encounter for asthma. This gives AUC an advantage over the other 5 performance metrics—accuracy, sensitivity, specificity, PPV, and NPV— whose values depend on the cutoff threshold used. For each classification algorithm, our automatic model selection method attempts to adjust all the related hyperparameters by testing many hyperparameter value combinations. To expedite the search, our method performs progressive sampling on the training set and uses test results on its subsets to quickly remove unpromising algorithms and hyperparameter value combinations. As a result, with no need to find near-optimal hyperparameter value combinations for almost all the algorithms, our method can return a good combination of the algorithm, feature selection technique, data balancing method, and hyperparameter values for building the final classification model. Compared with the Auto-WEKA automatic model selection method [46], our method can cut search time by 28-fold and model error rate by 11% simultaneously [45].

## Results

### Demographic Characteristics of Our Patient Cohort

Recall that each data instance targets a unique combination of an asthmatic patient and a year. Tables 2 and 3 exhibit the demographic characteristics of our patient cohort during 2005 to 2016 and 2017, respectively. The characteristics are relatively similar between the 2 periods. During 2005 to 2016 and 2017, about 3.59% (11,332/315,308) and 4.22% (812/19,256) of data instances linked to hospital encounters for asthma in the following year, respectively.

On the basis of chi-square 2-sample test, for both 2005 to 2016 and 2017 data, the data instances linked to future hospital encounters for asthma and those linked to no future hospital encounter for asthma showed the same distribution for long-acting beta2-agonist prescription (*P*=.67 for the 2005 to 2016 data and *P*=.11 for the 2017 data), mast cell stabilizer prescription (*P*=.29 for the 2005 to 2016 data and *P*>.99 for the 2017 data), allergic rhinitis occurrence (*P*=.38 for the 2005 to 2016 data and *P*=.13 for the 2017 data), and cystic fibrosis occurrence (*P*=.21 for the 2005 to 2016 data and *P*=.20 for the 2017 data) and, they showed different distributions for gender (*P*<.001 for the 2005 to 2016 data and *P*=.002 for the 2017 data), race (*P*<.001), ethnicity (*P*<.001), insurance category (*P*<.001), inhaled corticosteroid prescription (*P*<.001), inhaled steroid and rapid-onset long-acting beta2-agonist combination prescription (*P*<.001 for the 2005 to 2016 data and *P*=.002 for the 2017 data), leukotriene modifier prescription (*P*<.001), inhaled short-acting beta2-agonist prescription (*P*<.001), systemic corticosteroid prescription (*P*<.001), anxiety or depression occurrence (*P*<.001 for the 2005 to 2016 data and *P*=.002 for the 2017 data), bronchopulmonary dysplasia occurrence (*P*<.001 for the 2005 to 2016 data and *P*=.02 for the 2017 data), chronic obstructive pulmonary disease occurrence (*P*<.001), eczema occurrence (*P*<.001), gastroesophageal reflux occurrence (*P*<.001), obesity occurrence (*P*<.001 for the 2005 to 2016 data and *P*=.004 for the 2017 data), premature birth occurrence (*P*<.001), sleep apnea occurrence (*P*<.001), and smoking status (*P*<.001). For the data from 2005 to 2016, different distributions were shown for sinusitis occurrence (*P*=.006). For the 2017 data, the same distribution was shown for sinusitis occurrence (*P*=.91). On the basis of the Cochran-Armitage trend test [47], for both 2005 to 2016 and 2017 data, the data instances linked to future hospital encounters for asthma and those linked to no future hospital encounter for asthma showed different distributions for age (*P*<.001) and duration of asthma (*P*<.001).

**Table 2:** Demographic characteristics of the asthmatic patients at Intermountain Healthcare during 2005 to 2016.

Characteristics		Data instances (N=315,308), n (%)	Data instances linked to hospital encounters for asthma in the following year (N=11,332), n (%)	Data instances linked to no hospital encounter for asthma in the following year (N=303,976), n (%)
**Age (years)**				
	<6	37,826 (12.00)	3118 (27.52)	34,708 (11.42)
	6 to <18	53,162 (16.86)	2590 (22.86)	50,572 (16.64)
	18 to 65	177,439 (56.27)	5003 (44.15)	172,436 (56.73)
	65+	46,881 (14.87)	621 (5.48)	46,260 (15.22)
**Gender**				
	Male	127,217 (40.35)	5169 (45.61)	122,048 (40.15)
	Female	188,091 (59.65)	6163 (54.39)	181,928 (59.85)
**Race**				
	American Indian or Alaskan native	2509 (0.80)	214 (1.89)	2295 (0.76)
	Asian	2197 (0.70)	77 (0.68)	2120 (0.70)
	Black or African American	5751 (1.82)	460 (4.06)	5291 (1.74)
	Native Hawaiian or other Pacific Islander	4288 (1.36)	411 (3.63)	3877 (1.28)
	White	282,626 (89.63)	9420 (83.13)	273,206 (89.88)
	Unknown or not reported	17,937 (5.69)	750 (6.62)	17,187 (5.65)
**Ethnicity**				
	Hispanic	29,293 (9.29)	2279 (20.11)	27,014 (8.89)
	Non-Hispanic	252,599 (80.11)	8157 (71.98)	244,442 (80.41)
	Unknown or not reported	33,416 (10.60)	896 (7.91)	32,520 (10.70)
**Insurance**				
	Private	206,641 (65.54)	6192 (54.64)	200,449 (65.94)
	Public	80,154 (25.42)	3238 (28.57)	76,916 (25.30)
	Self-paid or charity	28,513 (9.04)	1902 (16.78)	26,611 (8.75)
**Duration of asthma (years)**				
	≤3	234,832 (74.48)	7666 (67.65)	227,166 (74.73)
	>3	80,476 (25.52)	3666 (32.35)	76,810 (25.27)
**Asthma medication prescription**				
	Inhaled corticosteroid	78,105 (24.77)	4539 (40.05)	73,566 (24.20)
	Inhaled steroid and rapid-onset long-acting beta2-agonist combination	44,992 (14.27)	2196 (19.38)	42,796 (14.08)
	Leukotriene modifier	35,507 (11.26)	2320 (20.47)	33,187 (10.92)
	Long-acting beta2-agonist	1813 (0.58)	69 (0.61)	1744 (0.57)
	Mast cell stabilizer	121 (0.04)	7 (0.06)	114 (0.04)
	Inhaled short-acting beta2-agonist	129,528 (41.08)	7545 (66.58)	121,983 (40.13)
	Systemic corticosteroid	136,642 (43.34)	7324 (64.63)	129,318 (42.54)
**Comorbidity**				
	Allergic rhinitis	4715 (1.50)	181 (1.60)	4534 (1.49)
	Anxiety or depression	56,961 (18.07)	1716 (15.14)	55,245 (18.17)
	Bronchopulmonary dysplasia	429 (0.14)	35 (0.31)	394 (0.13)
	Chronic obstructive pulmonary disease	12,887 (4.09)	391 (3.45)	12,496 (4.11)
	Cystic fibrosis	458 (0.15)	11 (0.10)	447 (0.15)
	Eczema	4927 (1.56)	443 (3.91)	4484 (1.48)
	Gastroesophageal reflux	56,196 (17.82)	1309 (11.55)	54,887 (18.06)
	Obesity	36,291 (11.51)	1076 (9.50)	35,215 (11.58)
	Premature birth	5542 (1.76)	440 (3.88)	5102 (1.68)
	Sinusitis	14,756 (4.68)	592 (5.22)	14,164 (4.66)
	Sleep apnea	20,892 (6.63)	471 (4.16)	20,421 (6.72)
**Smoking status**				
	Current smoker	35,551 (11.28)	1811 (15.98)	33,740 (11.10)
	Former smoker	19,304 (6.12)	569 (5.02)	18,735 (6.16)
	Never smoker or unknown	260,453 (82.60)	8952 (79.00)	251,501 (82.74)

**Table 3:** Demographic characteristics of the asthmatic patients at Intermountain Healthcare in 2017.

Characteristics		Data instances (N=19,256), n (%)	Data instances linked to hospital encounters for asthma in the following year (N=812), n (%)	Data instances linked to no hospital encounter for asthma in the following year (N=18,444), n (%)
**Age (years)**				
	<6	1877 (9.75)	199 (24.51)	1678 (9.10)
	6 to <18	3235 (16.80)	181 (22.29)	3054 (16.56)
	18 to 65	10,265 (53.31)	386 (47.54)	9879 (53.56)
	65+	3879 (20.14)	46 (5.67)	3833 (20.78)
**Gender**				
	Male	7816 (40.59)	373 (45.94)	7443 (40.35)
	Female	11,440 (59.41)	439 (54.06)	11,001 (59.65)
**Race**				
	American Indian or Alaskan native	159 (0.83)	13 (1.60)	146 (0.79)
	Asian	205 (1.06)	10 (1.23)	195 (1.06)
	Black or African American	403 (2.09)	42 (5.17)	361 (1.96)
	Native Hawaiian or other Pacific Islander	346 (1.80)	47 (5.79)	299 (1.62)
	White	17,706 (91.95)	681 (83.87)	17,025 (92.31)
	Unknown or not reported	437 (2.27)	19 (2.34)	418 (2.27)
**Ethnicity**				
	Hispanic	2212 (11.49)	192 (23.65)	2020 (10.95)
	Non-Hispanic	16,860 (87.56)	618 (76.11)	16,242 (88.06)
	Unknown or not reported	184 (0.96)	2 (0.25)	182 (0.99)
**Insurance**				
	Private	12,850 (66.73)	462 (56.90)	12,388 (67.17)
	Public	5128 (26.63)	208 (25.62)	4920 (26.68)
	Self-paid or charity	1278 (6.64)	142 (17.49)	1136 (6.16)
**Duration of asthma (years)**				
	≤3	11,133 (57.82)	423 (52.09)	10,710 (58.07)
	>3	8123 (42.18)	389 (47.91)	7734 (41.93)
**Asthma medication prescription**				
	Inhaled corticosteroid	7241 (37.60)	424 (52.22)	6817 (36.96)
	Inhaled steroid and rapid-onset long-acting beta2-agonist combination	4400 (22.85)	222 (27.34)	4178 (22.65)
	Leukotriene modifier	3573 (18.56)	209 (25.74)	3364 (18.24)
	Long-acting beta2-agonist	52 (0.27)	5 (0.62)	47 (0.25)
	Mast cell stabilizer	8 (0.04)	0 (0.00)	8 (0.04)
	Inhaled short-acting beta2-agonist	13,785 (71.59)	739 (91.01)	13,046 (70.73)
	Systemic corticosteroid	12,020 (62.42)	693 (85.34)	11,327 (61.41)
**Comorbidity**				
	Allergic rhinitis	392 (2.04)	10 (1.23)	382 (2.07)
	Anxiety or depression	3946 (20.49)	131 (16.13)	3815 (20.68)
	Bronchopulmonary dysplasia	15 (0.08)	3 (0.37)	12 (0.07)
	Chronic obstructive pulmonary disease	1056 (5.48)	23 (2.83)	1033 (5.60)
	Cystic fibrosis	95 (0.49)	1 (0.12)	94 (0.51)
	Eczema	307 (1.59)	34 (4.19)	273 (1.48)
	Gastroesophageal reflux	3548 (18.43)	71 (8.74)	3477 (18.85)
	Obesity	3505 (18.20)	116 (14.29)	3389 (18.37)
	Premature birth	476 (2.47)	41 (5.05)	435 (2.36)
	Sinusitis	780 (4.05)	34 (4.19)	746 (4.04)
	Sleep apnea	3003 (15.60)	78 (9.61)	2925 (15.86)
**Smoking status**				
	Current smoker	2391 (12.42)	146 (17.98)	2245 (12.17)
	Former smoker	2326 (12.08)	83 (10.22)	2243 (12.16)
	Never smoker or unknown	14,539 (75.50)	583 (71.80)	13,956 (75.67)

### Features and Classification Algorithm Used

After finishing the search process to maximize the AUC, our automatic model selection method [45] chose the XGBoost classification algorithm [43] and the hyperparameter values listed in Multimedia Appendix 1. XGBoost is based on decision trees and can deal with missing feature values naturally. As XGBoost only accepts numerical features as its inputs, each categorical feature was first converted into 1 or more binary features via one-hot encoding before being given to XGBoost. Our final model was constructed using XGBoost and the 142 features listed in the descending order of their importance values in the second table in Multimedia Appendix 1. Due to having no extra predictive power, the other features were automatically removed by XGBoost. As detailed in the book by Hastie et al [48], XGBoost automatically computed each feature’s importance value as the mean of such values across all decision trees in the XGBoost model. In each tree, the feature’s importance value was computed based on the performance improvement gained by the split at each internal node of the tree using the feature as the splitting variable, weighted by the number of data instances the node is responsible for.

### Performance Measures Achieved

**Figure 1:**
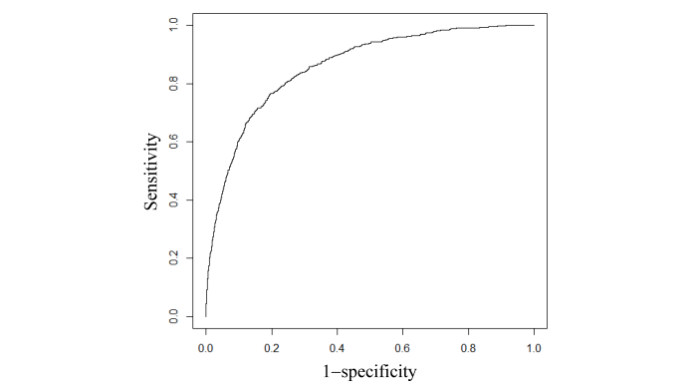
Our model’s receiver operating characteristic curve.

Our final model reached an AUC of 0.859 (95% CI 0.846-0.871). Figure 1 shows our final model’s receiver operating characteristic curve. Table 4 shows our final model’s performance metrics when differing top percentages of asthmatic patients with the highest predicted risk were used as the cutoff threshold for conducting binary classifications. When this threshold was at 10.00% (1926/19,256), our final model reached an accuracy of 90.31% (17,391/19,256; 95% CI 89.86-90.70), a sensitivity of 53.7% (436/812; 95% CI 50.12-57.18), a specificity of 91.93% (16,955/18,444; 95% CI 91.54-92.31), a PPV of 22.65% (436/1925; 95% CI 20.74-24.61), and an NPV of 97.83% (16,955/17,331; 95% CI 97.60-98.04). Table 5 shows the corresponding confusion matrix of our final model.

Recall that several features require more than 1 year of historical data to compute. If we exclude these features and use only those features computed on 1 year of historical data, the model’s AUC degrades to 0.849.

Without excluding the features that require more than 1 year of historical data to compute, the model trained on both asthmatic adults’ (age ≥18 years) and asthmatic children’s (age <18 years) data reached an AUC of 0.856 on asthmatic adults and an AUC of 0.830 on asthmatic children. In comparison, the model trained only on asthmatic adults’ data reached an AUC of 0.855 on asthmatic adults. The model trained only on asthmatic children’s data reached an AUC of 0.821 on asthmatic children.

If we used only the top 21 features listed in the second table in Multimedia Appendix 1 with an importance value ≥0.01 and excluded the other 121 features, the model’s AUC degraded from 0.859 to 0.855 (95% CI 0.842-0.867). When the cutoff threshold for conducting binary classification was set at the top 10.00% (1926/19,256) of asthmatic patients with the highest predicted risk, the model’s accuracy degraded from 90.31% (17,391/19,256) to 90.14% (17,357/19,256; 95% CI 89.74-90.58), sensitivity degraded from 53.7% (436/812) to 51.6% (419/812; 95% CI 48.02-55.24), specificity degraded from 91.93% (16,955/18,444) to 91.83% (16,938/18,444; 95% CI 91.43-92.24), PPV degraded from 22.65% (436/1925) to 21.77% (419/1925; 95% CI 20.03-23.68), and NPV degraded from 97.83% (16,955/17,331) to 97.73% (16,938/17,331; 95% CI 97.49-97.95).

**Table 4:** Our final model’s performance metrics when differing top percentages of asthmatic patients with the highest predicted risk were used as the cutoff threshold for conducting binary classification.

Top percentage of asthmatic patients with the highest predicted risk (%)	Accuracy (%)	Sensitivity (%)	Specificity (%)	Positive predictive value (%)	Negative predictive value (%)
1.00	95.89	13.05	99.53	55.21	96.30
2.00	95.54	20.81	98.83	43.90	96.59
3.00	95.00	26.23	98.03	36.92	96.79
4.00	94.48	32.02	97.23	33.77	97.01
5.00	93.84	36.21	96.38	30.56	97.17
6.00	93.19	40.39	95.52	28.40	97.33
7.00	92.53	44.33	94.65	26.73	97.48
8.00	91.85	48.15	93.77	25.39	97.62
9.00	91.09	51.11	92.85	23.95	97.73
10.00	90.31	53.69	91.93	22.65	97.83
15.00	86.44	67.00	87.29	18.84	98.36
20.00	81.95	73.15	82.34	15.42	98.58
25.00	77.41	78.57	77.36	13.25	98.80

**Table 5:** Our final model’s confusion matrix when the cutoff threshold for conducting binary classification was set at the top 10.00% (1926/19,256) of asthmatic patients with the highest predicted risk.

Class	Future hospital encounters for asthma, n	No future hospital encounter for asthma, n
Predicted future hospital encounters for asthma	436	1489
Predicted no future hospital encounter for asthma	376	16,955

## Discussion

### Principal Findings

We built a more accurate machine learning classification model to predict hospital encounters for asthma in the following year in asthmatic patients. Our final model achieved a higher AUC than what has been reported in the literature for this task [9-22]. After further refinement to improve its accuracy and to automatically explain its prediction results [49,50], our final model could be integrated into an electronic medical record system to guide care management allocation for asthmatic patients. This could better allocate a scarce and expensive resource and help improve asthma outcomes.

Asthma in adults is different from asthma in children. Our final model reached a higher AUC on asthmatic adults than on asthmatic children. More work is needed to understand the reason for this difference. In addition, more work is needed to improve the prediction accuracy on asthmatic children compared with asthmatic adults.

We considered 235 features in total, about 60% of which appeared in our final model. If a feature is unused by our final model, it does not necessarily mean that this feature has no predictive power. Rather, it only shows that this feature offers no extra predictive power on our specific dataset beyond what the features used in our final model have. On a larger dataset with more asthmatic patients, it is possible that some of the excluded features will provide extra predictive power. This is particularly true with features whose nontrivial values occur on only a small portion of asthmatic patients, such as a comorbidity with a low prevalence rate. When too few data instances take nontrivial values, the features’ predictive power may not appear.

In the second table in Multimedia Appendix 1, the 2 most important features, as well as several within the top 20, reflect overall instability of the patient’s asthma. The instability could derive from physiologic characteristics of the patient’s asthma, as reflected by the maximum blood eosinophil count, the maximum percentage of blood eosinophils, and the average respiratory rate. The instability could also result from treatment noncompliance, PCP changes, insurance changes, and socioeconomic issues for which data were unavailable.

### Comparison With Prior Work

Researchers have developed multiple models to predict inpatient stays and ED visits in asthmatic patients [9-22]. Table 6 compares our final model with these models, which include all relevant ones mentioned in Loymans et al’s recent systematic review [9]. None of these models obtained an AUC >0.81, whereas our final model’s AUC is 0.859. In other words, compared with our final model, each of these models reached an AUC lower by at least 0.049. Compared with prior model building, our model building assessed more candidate features with predictive power, adopted a more advanced classification algorithm, and used data from more asthmatic patients. All of these helped boost our final model’s accuracy. Our principle of considering extensive candidate features to help enhance the model’s accuracy is general and can be applied to other diseases and outcomes such as health care cost [51].

Except for Yurk et al’s model [17], all other prior models had a PPV ≤22% and a sensitivity ≤49%, which are lower than those achieved by our final model. Yurk et al’s model [17] obtained better sensitivity and PPV primarily because the model used a different prediction target: hospital encounters or ≥1 day lost because of reduced activities or missed work for asthma. This prediction target occurs for more than half of the asthmatic patients, making it relatively easy to predict. If the prediction target were changed to hospital encounters for asthma, a rarer outcome that is harder to predict, we would expect the sensitivity and PPV reached by Yurk et al’s model [17] to drop.

**Table 6:** A comparison of our final model and multiple prior models for predicting inpatient stays and emergency department visits in asthmatic patients.

Model	Prediction target	Classification algorithm	Features used in the model, n	Data instances, n	Area under the receiver operating characteristic curve	Sensitivity (%)	Specificity (%)	Positive predictive value (%)	Negative predictive value (%)
Our final model	Hospital encounters for asthma	Extreme gradient boosting	142	334,564	0.859	53.69	91.93	22.65	97.83
Loymans et al [10]	Asthma exacerbation	Logistic regression	7	611	0.8	—^a^	—	—	—
Schatz et al [11]	Inpatient stay for asthma in children	Logistic regression	5	4197	0.781	43.9	89.8	5.6	99.1
Schatz et al [11]	Inpatient stay for asthma in adults	Logistic regression	3	6904	0.712	44.9	87.0	3.9	99.3
Eisner et al [12]	Inpatient stay for asthma	Logistic regression	1	2858	0.689	—	—	—	—
Eisner et al [12]	ED^b^ visit for asthma	Logistic regression	3	2415	0.751	—	—	—	—
Sato et al [13]	Severe asthma exacerbation	Classification and regression tree	3	78	0.625	—	—	—	—
Miller et al [15]	Hospital encounters for asthma	Logistic regression	17	2821	0.81	—	—	—	—
Yurk et al [17]	Hospital encounters or lost day for asthma	Logistic regression	11	4888	0.78	77	63	82	56
Lieu et al [18]	Inpatient stay for asthma	Proportional hazards regression	7	16,520	0.79	—	—	—	—
Lieu et al [18]	ED visit for asthma	Proportional hazards regression	7	16,520	0.69	—	—	—	—
Lieu et al [19]	Hospital encounters for asthma	Classification and regression tree	4	7141	—	49.0	83.6	18.5	—
Schatz et al [20]	Hospital encounters for asthma	Logistic regression	4	14,893	0.614	25.4	92.0	22.0	93.2
Forno et al [22]	Severe asthma exacerbation	Scoring	17	615	0.75	—	—	—	—

^a^The performance measure is not reported in the original paper describing the model.

^b^ED: emergency department.

### Considerations Regarding Potential Clinical Use

Despite being more accurate than the prior ones, our final model still reached a relatively low PPV of 22.65% (436/1925). However, this does not prevent our final model from being clinically useful because of the following reasons:

- A PPV of 22.65% is reasonably good for identifying high-risk asthmatic patients as candidates for receiving relatively inexpensive preventive interventions. Furthermore, 4 examples of such interventions are teaching the patient how to correctly use an asthma inhaler, teaching the patient how to correctly use a peak flow meter and giving it to the patient to use at home for self-monitoring, training the patient to keep an environmental trigger diary, and arranging for a nurse to make additional follow-up phone calls with the patient.
- The PPV depends highly on the outcome’s prevalence rate [52]. A relatively rare outcome, such as future hospital encounters for asthma, will occur in only a finite number of patients. Hence, most patients projected to have the outcome will inevitably turn out to not have the outcome, causing even a good predictive model to have a low PPV [52]. For such an outcome, sensitivity is more important than PPV for assessing the model’s performance and potential clinical impact. As shown in Table 4, by setting the cutoff threshold for conducting binary classification at the top 10.00% (1926/19,256) of patients with the highest predicted risk, our final model has already captured 53.7% (436/812) of the asthmatic patients who will incur future hospital encounters for asthma. If one is willing to increase the cutoff threshold to the top 25.00% (4814/19,256) of patients with the highest predicted risk, our final model would have captured 78.6% (638/812) of the asthmatic patients who will incur future hospital encounters for asthma, even though the PPV is only 13.25% (638/4814).
- Proprietary models with performance measures similar to those of the previously published models are being used at health care systems such as Intermountain Healthcare, University of Washington Medicine, and Kaiser Permanente Northern California [18] for allocating preventive interventions. Our final model is an improvement over those models. Table 6 shows that compared with the previously published models, our final model reached a sensitivity higher by 4.69% or more. If we could use our final model to find 4.69% more asthmatic patients who will incur future hospital encounters for asthma and enroll them in care management, we could improve outcomes and avoid up to 9239 inpatient stays and 33,768 ED visits each year [1,4-7]. Supporting the importance of relatively small improvements in the model’s performance measures, Razavian et al [53] showed that by reaching a gain of 0.05 in AUC (from 0.75 to 0.8) and a PPV of 15%, a large health insurance company such as Independence Blue Cross would be willing to deploy a new predictive model to appropriately allocate preventive interventions.

Our final model used 142 features. Reducing features used in the model could ease its clinical deployment. For this, one could use the top few features with the highest importance values (eg, ≥0.01) and exclude the others, if one is willing to accept a not-too-big degrade of model accuracy. Ideally, one should first assess the features’ importance values on a dataset from the target health care system before deciding which features should be kept for that system. A feature’s importance value varies across different health care systems. A feature with a low importance value on the Intermountain Healthcare dataset might have a decent importance value on a dataset from another health care system. Similar to the case with many other complex machine learning models, an XGBoost model using a nontrivial number of features is difficult to interpret globally. As an interesting area for future work, we are in the process of investigating using the automatic explanation approach described in our prior papers [49,50] to automatically explain our final XGBoost model’s prediction results on individual asthmatic patients.

Our final model was built using the XGBoost classification algorithm [43]. For binary classification with 2 unbalanced classes, XGBoost uses a hyperparameter scale_pos_weight to control the balance of the weights for the positive and negative classes [54]. One could set scale_pos_weight to the ratio of the number of negative data instances to the number of positive data instances [54], although the optimal value of scale_pos_weight often deviates from this value by a degree varying by the specific dataset. In our case, to maximize the model’s AUC, our automatic model selection method [45] did a search of possible hyperparameter values and eventually set scale_pos_weight to a nondefault value to balance the 2 classes of future hospital encounters for asthma or not [55]. This has the side effect of making the model’s predicted probabilities of incurring future hospital encounters for asthma very small and unaligned with the actual probabilities [55]. This side effect does not prevent us from selecting the top few percentage of asthmatic patients with the highest predicted risk as candidates for receiving care management or other preventive interventions. To avoid this side effect, we could set scale_pos_weight to its default value of 1, without balancing the 2 classes. However, that would degrade the model’s AUC from 0.859 to 0.849 (95% CI 0.836-0.862).

### Limitations

This study has several limitations, all of which provide interesting areas for future work:

- We had no access to medication claim data. Consequently, we were unable to use as features the following major risk factors for hospital encounters for asthma in asthmatic patients: medication compliance reflected in refill frequency, the asthma medication ratio [56], the dose of inhaled corticosteroids [33], and the step number of the stepwise approach for managing asthma [33,57]. We are in the process of obtaining an asthmatic patient dataset from Kaiser Permanente Southern California including these attributes [58], so that we can investigate how much gain in prediction accuracy they can bring.
- Besides those considered in the study, other features could also help boost model accuracy. Our dataset missed some of these features, such as pulmonary function test results. An example of pulmonary function test results is the ratio of the forced expiratory volume in 1 second to the forced vital capacity, a known risk factor for hospital encounters for asthma in asthmatic patients. It would be interesting to find new predictive features from, but not limited to, the attributes available in our dataset.
- Our study considered only structured data and non–deep-learning machine learning classification algorithms. Adding features extracted from unstructured clinical notes and using deep learning may further improve the model’s accuracy [50,58].
- Our dataset included no information on the patients’ health care use at non–Intermountain Healthcare facilities. As a result, we computed features using incomplete clinical and administrative data of the patients [59-62]. In addition, instead of taking hospital encounters for asthma anywhere as the prediction target, we had to restrict it to hospital encounters for asthma at Intermountain Healthcare. It would be interesting to investigate how the model’s accuracy would change if more complete clinical and administrative data of the patients are available [63].
- Our study used data from 1 health care system and did not assess our results’ generalizability. After obtaining the asthmatic patient dataset from Kaiser Permanente Southern California, we plan to evaluate our final model’s performance on that dataset and explore the process of customizing models to features available in specific datasets as part of the approach to generalization.

### Conclusions

Our final model improves the state of the art for predicting hospital encounters for asthma in asthmatic patients. In particular, our final model reached an AUC of 0.859, which is higher than those previously reported in the literature for this task by ≥0.049. After further refinement, our final model could be integrated into an electronic medical record system to guide allocation of scarce care management resources for asthmatic patients. This could help improve the value equation for asthma care by improving asthma outcomes while also decreasing resource use and cost.

## Appendix

Multimedia Appendix 1 The candidate features.

## Abbreviations

AUC: area under the receiver operating characteristic curve
ED: emergency department
FN: false negative
FP: false positive
ICD-9: International Classification of Diseases, Ninth Revision
ICD-10: International Classification of Diseases, Tenth Revision
NPV: negative predictive value
PCP: primary care provider
PPV: positive predictive value
TN: true negative
TP: true positive
Weka: Waikato Environment for Knowledge Analysis
XGBoost: extreme gradient boosting

## References

[1] Moorman JE, Akinbami LJ, Bailey CM, Zahran HS, King ME, Johnson CA, Liu X (2012). National surveillance of asthma: United States, 2001-2010. Vital Health Stat 3.

[2] Nurmagambetov T, Kuwahara R, Garbe P (2018). The economic burden of asthma in the United States, 2008-2013. Ann Am Thorac Soc.

[3] Mays GP, Claxton G, White J (2004). Managed care rebound? Recent changes in health plans' cost containment strategies. Health Aff (Millwood).

[4] Caloyeras JP, Liu H, Exum E, Broderick M, Mattke S (2014). Managing manifest diseases, but not health risks, saved PepsiCo money over seven years. Health Aff (Millwood).

[5] Greineder DK, Loane KC, Parks P (1999). A randomized controlled trial of a pediatric asthma outreach program. J Allergy Clin Immunol.

[6] Kelly CS, Morrow AL, Shults J, Nakas N, Strope GL, Adelman RD (2000). Outcomes evaluation of a comprehensive intervention program for asthmatic children enrolled in medicaid. Pediatrics.

[7] Axelrod RC, Zimbro KS, Chetney RR, Sabol J, Ainsworth VJ (2001). A disease management program utilizing life coaches for children with asthma. J Clin Outcomes Manag.

[8] Axelrod RC, Vogel D (2003). Predictive modeling in health plans. Dis Manag Health Outcomes.

[9] Loymans RJ, Debray TP, Honkoop PJ, Termeer EH, Snoeck-Stroband JB, Schermer TR, Assendelft WJ, Timp M, Chung KF, Sousa AR, Sont JK, Sterk PJ, Reddel HK, Riet GT (2018). Exacerbations in adults with asthma: a systematic review and external validation of prediction models. J Allergy Clin Immunol Pract.

[10] Loymans RJ, Honkoop PJ, Termeer EH, Snoeck-Stroband JB, Assendelft WJ, Schermer TR, Chung KF, Sousa AR, Sterk PJ, Reddel HK, Sont JK, Riet GT (2016). Identifying patients at risk for severe exacerbations of asthma: development and external validation of a multivariable prediction model. Thorax.

[11] Schatz M, Cook EF, Joshua A, Petitti D (2003). Risk factors for asthma hospitalizations in a managed care organization: development of a clinical prediction rule. Am J Manag Care.

[12] Eisner MD, Yegin A, Trzaskoma B (2012). Severity of asthma score predicts clinical outcomes in patients with moderate to severe persistent asthma. Chest.

[13] Sato R, Tomita K, Sano H, Ichihashi H, Yamagata S, Sano A, Yamagata T, Miyara T, Iwanaga T, Muraki M, Tohda Y (2009). The strategy for predicting future exacerbation of asthma using a combination of the Asthma Control Test and lung function test. J Asthma.

[14] Osborne ML, Pedula KL, O'Hollaren M, Ettinger KM, Stibolt T, Buist AS, Vollmer WM (2007). Assessing future need for acute care in adult asthmatics: the Profile of Asthma Risk Study: a prospective health maintenance organization-based study. Chest.

[15] Miller MK, Lee JH, Blanc PD, Pasta DJ, Gujrathi S, Barron H, Wenzel SE, Weiss ST, TENOR Study Group (2006). TENOR risk score predicts healthcare in adults with severe or difficult-to-treat asthma. Eur Respir J.

[16] Peters D, Chen C, Markson LE, Allen-Ramey FC, Vollmer WM (2006). Using an asthma control questionnaire and administrative data to predict health-care utilization. Chest.

[17] Yurk RA, Diette GB, Skinner EA, Dominici F, Clark RD, Steinwachs DM, Wu AW (2004). Predicting patient-reported asthma outcomes for adults in managed care. Am J Manag Care.

[18] Lieu TA, Quesenberry CP, Sorel ME, Mendoza GR, Leong AB (1998). Computer-based models to identify high-risk children with asthma. Am J Respir Crit Care Med.

[19] Lieu TA, Capra AM, Quesenberry CP, Mendoza GR, Mazar M (1999). Computer-based models to identify high-risk adults with asthma: is the glass half empty of half full?. J Asthma.

[20] Schatz M, Nakahiro R, Jones CH, Roth RM, Joshua A, Petitti D (2004). Asthma population management: development and validation of a practical 3-level risk stratification scheme. Am J Manag Care.

[21] Grana J, Preston S, McDermott PD, Hanchak NA (1997). The use of administrative data to risk-stratify asthmatic patients. Am J Med Qual.

[22] Forno E, Fuhlbrigge A, Soto-Quirós ME, Avila L, Raby BA, Brehm J, Sylvia JM, Weiss ST, Celedón JC (2010). Risk factors and predictive clinical scores for asthma exacerbations in childhood. Chest.

[23] Desai JR, Wu P, Nichols GA, Lieu TA, O'Connor PJ (2012). Diabetes and asthma case identification, validation, and representativeness when using electronic health data to construct registries for comparative effectiveness and epidemiologic research. Med Care.

[24] Wakefield DB, Cloutier MM (2006). Modifications to HEDIS and CSTE algorithms improve case recognition of pediatric asthma. Pediatr Pulmonol.

[25] Luo G, Stone BL, Sakaguchi F, Sheng X, Murtaugh MA (2015). Using computational approaches to improve risk-stratified patient management: rationale and methods. JMIR Res Protoc.

[26] Luo G, Sward K (2017). A roadmap for optimizing asthma care management via computational approaches. JMIR Med Inform.

[27] Puranik S, Forno E, Bush A, Celedón JC (2017). Predicting severe asthma exacerbations in children. Am J Respir Crit Care Med.

[28] Buelo A, McLean S, Julious S, Flores-Kim J, Bush A, Henderson J, Paton JY, Sheikh A, Shields M, Pinnock H, ARC Group (2018). At-risk children with asthma (ARC): a systematic review. Thorax.

[29] Greenberg S (2013). Asthma exacerbations: predisposing factors and prediction rules. Curr Opin Allergy Clin Immunol.

[30] Fleming L (2018). Asthma exacerbation prediction: recent insights. Curr Opin Allergy Clin Immunol.

[31] Purdey S, Huntley A (2013). Predicting and preventing avoidable hospital admissions: a review. J R Coll Physicians Edinb.

[32] Ledford DK, Lockey RF (2013). Asthma and comorbidities. Curr Opin Allergy Clin Immunol.

[33] Blakey JD, Price DB, Pizzichini E, Popov TA, Dimitrov BD, Postma DS, Josephs LK, Kaplan A, Papi A, Kerkhof M, Hillyer EV, Chisholm A, Thomas M (2017). Identifying risk of future asthma attacks using UK medical record data: a respiratory effectiveness group initiative. J Allergy Clin Immunol Pract.

[34] Das LT, Abramson EL, Stone AE, Kondrich JE, Kern LM, Grinspan ZM (2017). Predicting frequent emergency department visits among children with asthma using EHR data. Pediatr Pulmonol.

[35] Quan H, Sundararajan V, Halfon P, Fong A, Burnand B, Luthi JC, Saunders LD, Beck CA, Feasby TE, Ghali WA (2005). Coding algorithms for defining comorbidities in ICD-9-CM and ICD-10 administrative data. Med Care.

[36] Wallace E, Stuart E, Vaughan N, Bennett K, Fahey T, Smith SM (2014). Risk prediction models to predict emergency hospital admission in community-dwelling adults: a systematic review. Med Care.

[37] (2017). ICPSR - University of Michigan.

[38] (2019). US Health Literacy Scores.

[39] Singh GK (2003). Area deprivation and widening inequalities in US mortality, 1969-1998. Am J Public Health.

[40] (2019). Guinness World Records.

[41] Steyerberg EW (2009). Clinical Prediction Models: A Practical Approach to Development, Validation, and Updating.

[42] Witten IH, Frank E, Hall MA, Pal CJ (2016). Data Mining: Practical Machine Learning Tools and Techniques. Fourth Edition.

[43] Chen T, Guestrin C (2016). XGBoost: A Scalable Tree Boosting System. Proceedings of the 22nd ACM SIGKDD International Conference on Knowledge Discovery and Data Mining.

[44] (2019). XGBoost Documentation.

[45] Zeng X, Luo G (2017). Progressive sampling-based Bayesian optimization for efficient and automatic machine learning model selection. Health Inf Sci Syst.

[46] Thornton C, Hutter F, Hoos HH, Leyton-Brown K (2013). Auto-WEKA: Combined Selection and Hyperparameter Optimization of Classification Algorithms. Proceedings of the 19th ACM SIGKDD International Conference on Knowledge Discovery and Data mining.

[47] Agresti A (2012). Categorical Data Analysis. Third Edition.

[48] Hastie T, Tibshirani R, Friedman J (2016). The Elements of Statistical Learning: Data Mining, Inference, and Prediction. Second Edition.

[49] Luo G (2016). Automatically explaining machine learning prediction results: a demonstration on type 2 diabetes risk prediction. Health Inf Sci Syst.

[50] Luo G (2019). A roadmap for semi-automatically extracting predictive and clinically meaningful temporal features from medical data for predictive modeling. Glob Transit.

[51] Goldstein BA, Navar AM, Pencina MJ, Ioannidis JPA (2017). Opportunities and challenges in developing risk prediction models with electronic health records data: a systematic review. J Am Med Inform Assoc.

[52] Ranganathan P, Aggarwal R (2018). Common pitfalls in statistical analysis: understanding the properties of diagnostic tests - Part 1. Perspect Clin Res.

[53] Razavian N, Blecker S, Schmidt AM, Smith-McLallen A, Nigam S, Sontag D (2015). Population-level prediction of type 2 diabetes from claims data and analysis of risk factors. Big Data.

[54] (2019). XGBoost Documentation.

[55] (2019). XGBoost Documentation.

[56] Andrews AL, Simpson AN, Basco WT Jr, Teufel RJ 2nd (2013). Asthma medication ratio predicts emergency department visits and hospitalizations in children with asthma. Medicare Medicaid Res Rev.

[57] National Asthma Education and Prevention Program (2007). National Heart, Lung, and Blood Institute (NHLBI) - NIH.

[58] Luo G, Stone BL, Koebnick C, He S, Au DH, Sheng X, Murtaugh MA, Sward KA, Schatz M, Zeiger RS, Davidson GH, Nkoy FL (2019). Using temporal features to provide data-driven clinical early warnings for chronic obstructive pulmonary disease and asthma care management: protocol for a secondary analysis. JMIR Res Protoc.

[59] Bourgeois FC, Olson KL, Mandl KD (2010). Patients treated at multiple acute health care facilities: quantifying information fragmentation. Arch Intern Med.

[60] Finnell JT, Overhage JM, Grannis S (2011). All health care is not local: an evaluation of the distribution of emergency department care delivered in Indiana. AMIA Annu Symp Proc.

[61] Luo G, Tarczy-Hornoch P, Wilcox AB, Lee ES (2018). Identifying patients who are likely to receive most of their care from a specific health care system: demonstration via secondary analysis. JMIR Med Inform.

[62] Kern LM, Grinspan Z, Shapiro JS, Kaushal R (2017). Patients' use of multiple hospitals in a major US city: implications for population management. Popul Health Manag.

[63] Samuels-Kalow ME, Faridi MK, Espinola JA, Klig JE, Camargo CA Jr (2018). Comparing statewide and single-center data to predict high-frequency emergency department utilization among patients with asthma exacerbation. Acad Emerg Med.

