# Supplementary material for: Developing a Model to Predict Hospital Encounters for Asthma in Asthmatic Patients: Secondary Analysis
Source: JMIR Med Inform. 2020 Jan 21;8(1):e16080. doi: 10.2196/16080 (PMC7001050; doi:10.2196/16080)
Supplement: Multimedia Appendix 1 [file medinform_v8i1e16080_app1.docx]

**Appendix**

**Table 1**. The candidate features.

| Category | Features |
| --- | --- |
| Patient demographics features | Age; gender; race; ethnicity (Hispanic or non-Hispanic); marital status (divorced, married, partnered, separated, single, or widowed); language; and religion. |
| Features reflecting properties of the area related to the five-digit zip code of the patient’s home address | The area’s population size; black population percentage; Hispanic population percentage; white population percentage; estimated number of households; average house value; average household income; estimated average number of people per household; average elevation; and the 2003 rural-urban continuum code, which is a number between 1 (most urban) and 9 (most rural) reflecting rurality [37]. Except for the last two, all of these features were derived from 2010 census data. |
| Features reflecting properties of the census block group where the patient resides | The block group’s number of employed people 16 and older; percentage of employed people 16 and older in a white-collar occupation; percentage of households with >1 person per room; percentage of households that are owner-occupied; percentage of single-parent households with dependents <18 years old; percentage of occupied housing units without complete plumbing; percentage of households without a phone; percentage of households without a motor vehicle; income disparity measure = log(the number of households with median income < US $15,000 / the number of households with median income > US $75,000); number of civilian labor force 16 and older; median family income; median home value; median monthly mortgage payment; median monthly rent payment; percentage of families below 150% of the federal poverty level; percentage of families below the federal poverty level; percentage of population 25 and older with < 9 years of education; percentage of population 25 and older with a high school diploma or higher education; combined population of each of the census blocks within the block group that qualifies as rural under the 2013 US Census; number of families; number of households; number of occupied households; size of the population 25 and older; population size; number of single-parent households; percentage of the civilian labor force 16 and older that is unemployed; combined population of each of the census blocks within the block group that qualifies as urban under the 2013 US census; national health literacy score developed by the University of North Carolina at Chapel Hill [38]; Singh’s area deprivation index measuring the neighborhood’s socioeconomic deprivation [39]; indicator of whether the urban population is larger than the rural population; and rural/urban status (rural, urban, or mixed urban/rural). Except for the last four, all of these features were based on the US Census 2013 American Community Survey five-year rolling averages. The last three features were based on 2013 US census data. |
| Laboratory test-related features | The number of laboratory tests; the number of laboratory tests with abnormal results; the number of days since having the last laboratory test; the maximum blood eosinophil count; the maximum percentage of blood eosinophils; the maximum total serum immunoglobulin E (IgE) level; whether the maximum total serum IgE level is abnormally high; and whether an IgE test was done. |
| Vital sign features | The maximum diastolic blood pressure; the average diastolic blood pressure; the maximum systolic blood pressure; the average systolic blood pressure; the maximum heart rate; the average heart rate; the maximum respiratory rate; the average respiratory rate; the maximum temperature; the average temperature; the minimum peripheral capillary oxygen saturation (SpO_2_); the average SpO_2_; the maximum body mass index (BMI); the change of BMI in percentage defined as (the last recorded BMI / the first recorded BMI - 1) × 100%; and the change of weight in percentage defined as (the last recorded weight / the first recorded weight - 1) × 100%. |
| Diagnosis-related features computed from ICD-9 and ICD-10 diagnosis codes only | The duration of asthma defined as the number of years for which the patient had asthma; the number of ICD-9 and ICD-10 diagnosis codes; chronic obstructive pulmonary disease; the duration of chronic obstructive pulmonary disease defined as the number of years for which the patient had this disease; ischemic heart disease; allergic rhinitis; gastroesophageal reflux; esophagitis; anxiety or depression; eczema; sleep apnea; obesity; gastrostomy tube; upper respiratory tract infection; Alzheimer’s or Parkinson’s disease; bronchiolitis; bronchopulmonary dysplasia; cystic fibrosis; decreased tone; increased tone; pneumonia; premature birth; vocal cord dysfunction; immunoglobulin A (IgA) deficiency; psoriasis; anaphylaxis; vasculitis; cirrhosis; inflammatory bowel disease; gastrointestinal bleeding; gastrointestinal obstruction; breathing abnormality like dyspnea; substance use; mental disorder; pregnancy; vitamin D deficiency; folate deficiency; myocardial infarction; congestive heart failure; peripheral vascular disease; cerebrovascular disease; dementia; rheumatic disease; peptic ulcer disease; mild liver disease; diabetes without chronic complication; diabetes with chronic complication; hemiplegia or paraplegia; renal disease; malignancy; moderate or severe liver disease; metastatic solid tumor; and acquired immunodeficiency syndrome/human immunodeficiency virus. |
| Diagnosis-related features computed simultaneously from ICD-9 and ICD-10 diagnosis codes, as well as Current Procedural Terminology (CPT) and Healthcare Common Procedure Coding System (HCPCS) procedure codes | Cataract; and sinusitis. |
| Diagnosis-related feature computed simultaneously from ICD-9 and ICD-10 diagnosis codes, as well as ICD-9 and ICD-10 procedure codes | Tracheostomy. |
| Diagnosis-related feature computed simultaneously from ICD-9 and ICD-10 diagnosis codes, as well as clinical assessment results | The patient’s smoking status (current smoker, former smoker, or never smoker or unknown). |
| Medication-related features | The number of medication orders; the total number of medications in all of the medication orders; the total number of distinct medications in all of the medication orders; the total number of refills allowed in all of the medication orders; the total number of units ordered in all of the medication orders; the number of asthma medication orders; the total number of medications in all of the asthma medication orders; the total number of distinct medications in all of the asthma medication orders; the total number of refills allowed in all of the asthma medication orders; the total number of units of asthma medications ordered; the total number of short-acting beta-2 agonists ordered; the total number of units of short-acting beta-2 agonists ordered; the total number of refills allowed in all of the short-acting beta-2 agonist orders; the total number of systemic corticosteroids ordered; the total number of units of systemic corticosteroids ordered; the total number of refills allowed in all of the systemic corticosteroid orders; the total number of asthma reliever medications ordered that are neither systemic corticosteroids nor short-acting beta-2 agonists; the total number of units of asthma reliever medications ordered that are neither systemic corticosteroids nor short-acting beta-2 agonists; the total number of inhaled corticosteroids ordered; the total number of units of inhaled corticosteroids ordered; the total number of refills allowed in all of the inhaled corticosteroid orders; the total number of mast cell stabilizers ordered; the total number of units of mast cell stabilizers ordered; the total number of refills allowed in all of the mast cell stabilizer orders; the total number of nonsteroidal anti-inflammatory drugs (NSAIDs) ordered; the total number of units of NSAIDs ordered; the total number of refills allowed in all of the NSAID orders; the total number of antihistamines ordered; the total number of units of antihistamines ordered; the total number of refills allowed in all of the antihistamine orders; the total number of allergen immunotherapy medications ordered; the total number of nasal steroid sprays ordered; the total number of units of nasal steroid sprays ordered; the total number of refills allowed in all of the nasal steroid spray orders; the total number of beta blockers ordered; the total number of units of beta blockers ordered; the total number of refills allowed in all of the beta blocker orders; the total number of statins ordered; the total number of units of statins ordered; the total number of refills allowed in all of the statin orders; whether spacer was used; and whether nebulizer was used. |
| Insurance-related features | The primary payer’s insurance category (Medicaid, Medicare, Intermountain Healthcare’s own health insurance plan SelectHealth, other private insurance, or self-paid or charity) at the patient’s last visit; the number of insurances of the patient at the last visit; and the number of distinct primary payers across all of the patient’s visits. |
| Visit type-related features for the patient | The number of outpatient visits; the number of outpatient visits with a primary diagnosis of asthma; the number of outpatient visits to the patient’s PCP; the number of outpatient visits to specialists; the number of outpatient visits to allergists and immunologists; the number of ED visits; the length of stay of the last ED visit; the average length of stay of an ED visit; the number of inpatient stays; the total length of all of the inpatient stays; the average length of an inpatient stay; the number of admissions to intensive care; the length of the last intensive care unit stay; the average length of an intensive care unit stay; the last visit’s admission type (emergency, urgent, elective, or trauma); the most emergent one among all of the visits’ admission types; and the number of major visits for asthma. |
| Features related to appointment scheduling and visit status | The number of no shows; the number of cancelled appointments; the number of visits that were referred; the day of the week at the last ED visit’s admission time; the admit hour of the last ED visit; the discharge disposition location (home, left against medical advice, or other non-home location) of the last visit; the time between making the request and the actual visit of the last visit reflecting the request’s urgency; the shortest time between making the request and the actual visit among all of the visits; the number of days since the last inpatient stay; whether the last inpatient stay was through the ED; the number of days since the last outpatient visit; the number of days since the last ED visit; the number of times the patient left against medical advice; and the acuity level (resuscitation, emergent, urgent, semi-urgent, or non-urgent) of the last ED visit. |
| Features reflecting care continuity of the patient | The number of distinct EDs the patient visited; the number of distinct providers seen in outpatient visits; the number of distinct PCPs of the patient; the number of distinct medication prescribers; the number of distinct asthma medication prescribers; whether the patient was homeless; whether the patient had no phone number; and the number of distinct addresses the patient had, reflecting the number of times the patient moved. |
| Procedure-related features | The number of ICD-9 and ICD-10 procedure codes; the number of CPT/HCPCS procedure codes; the number of CPT/HCPCS procedure codes for influenza vaccination; the number of HCPCS procedure codes for home oxygen therapy; the number of CPT procedure codes for pulmonary function tests; the number of CPT procedure codes for the fractional exhaled nitric oxide (FeNO) test; and mechanical ventilation shown by ICD-9 and ICD-10 procedure codes. |
| Radiology-related feature | The number of chest X-ray exams. |
| Allergy features | Whether the patient had any drug or material allergy; whether the patient had any environmental allergy; whether the patient had any food allergy; and the number of allergies of the patient. |
| Clinical assessment-related feature | The number of times the patient was assessed to be confused. |
| Provider features | We considered several features of the patient’s current PCP defined as the patient’s PCP known at the patient’s last clinic visit. These features include: whether the patient and the PCP are of the same gender; the PCP’s age; whether the PCP is a preferred provider of Intermountain Healthcare’s health insurance plan SelectHealth; the level of affiliation that the PCP has with Intermountain Healthcare (independent practitioner, employed by an Intermountain Healthcare hospital, employed by the Intermountain Medical Group managing Intermountain Healthcare’s clinics, or non-credentialed provider); the PCP’s primary specialty; the PCP’s primary profession type (Doctor of Medicine, Doctor of Osteopathic Medicine, nurse practitioner, family nurse practitioner, advanced practice registered nurse, physician assistant, or other); the number of asthmatic patients of the PCP; and the proportion who incurred hospital encounters for asthma in the index year out of all asthmatic patients of the PCP in the year before. |
| Facility features | The ellipsoid great circle distance between the patient’s home and the closest ED, which was computed based on the longitude and latitude coordinates of the ED location and the five-digit zip code of the patient’s home address; and the ellipsoid great circle distance between the patient’s home and the patient’s current PCP’s office, which was computed based on the longitude and latitude coordinates of the PCP’s office location and the five-digit zip code of the patient’s home address. |

**Table 2**. The features adopted in our final model and their importance values.

| Rank | Feature | Importance based on the feature’s fractional contribution to the model |
| --- | --- | --- |
| 1 | The number of major visits for asthma | 0.1413 |
| 2 | The total number of units of systemic corticosteroids ordered | 0.1241 |
| 3 | The number of days since the last ED visit | 0.0787 |
| 4 | Age | 0.0586 |
| 5 | The last visit’s admission type = elective | 0.0515 |
| 6 | Duration of asthma | 0.0482 |
| 7 | The number of ED visits | 0.0363 |
| 8 | The total number of units of short-acting beta-2 agonists ordered | 0.0327 |
| 9 | The total number of short-acting beta-2 agonists ordered | 0.0251 |
| 10 | The total number of systemic corticosteroids ordered | 0.0204 |
| 11 | The maximum blood eosinophil count | 0.0178 |
| 12 | The maximum percentage of blood eosinophils | 0.0177 |
| 13 | The number of ICD-9 and ICD-10 procedure codes | 0.0173 |
| 14 | The number of distinct asthma medication prescribers | 0.0167 |
| 15 | The average respiratory rate | 0.0139 |
| 16 | The average heart rate | 0.0129 |
| 17 | The total number of units ordered in all of the medication orders | 0.0128 |
| 18 | The proportion who incurred hospital encounters for asthma in the index year out of all asthmatic patients of the PCP in the year before | 0.0123 |
| 19 | Ethnicity | 0.0118 |
| 20 | Whether nebulizer was used | 0.0110 |
| 21 | The time between making the request and the actual visit of the last visit | 0.0109 |
| 22 | The number of asthma medication orders | 0.0077 |
| 23 | The number of ICD-9 and ICD-10 diagnosis codes | 0.0076 |
| 24 | The total number of distinct medications in all of the medication orders | 0.0075 |
| 25 | The total number of units of asthma medications ordered | 0.0072 |
| 26 | The block group’s national health literacy score | 0.0068 |
| 27 | The total number of distinct medications in all of the asthma medication orders | 0.0061 |
| 28 | The block group’s median family income | 0.0059 |
| 29 | Marital status = married | 0.0059 |
| 30 | The number of outpatient visits | 0.0057 |
| 31 | The percentage of families below 150% of the federal poverty level in the block group | 0.0049 |
| 32 | The total number of medications in all of the medication orders | 0.0049 |
| 33 | The maximum BMI | 0.0048 |
| 34 | The shortest time between making the request and the actual visit among all of the visits | 0.0046 |
| 35 | The number of laboratory tests with abnormal results | 0.0042 |
| 36 | The number of distinct providers seen in outpatient visits | 0.0038 |
| 37 | The average diastolic blood pressure | 0.0035 |
| 38 | The ellipsoid great circle distance between the patient’s home and the closest ED | 0.0034 |
| 39 | The change of BMI in percentage | 0.0034 |
| 40 | The total number of units of inhaled corticosteroids ordered | 0.0034 |
| 41 | Singh’s area deprivation index of the block group | 0.0034 |
| 42 | The number of insurances of the patient at the last visit | 0.0033 |
| 43 | The area’s black population percentage | 0.0032 |
| 44 | Race = white | 0.0028 |
| 45 | The average length of an inpatient stay | 0.0028 |
| 46 | The percentage of population 25 and older with a high school diploma or higher education in the block group | 0.0028 |
| 47 | The block group’s income disparity measure | 0.0028 |
| 48 | The area’s white population percentage | 0.0027 |
| 49 | The percentage of employed people 16 and older in the block group who are in a white-collar occupation | 0.0027 |
| 50 | The area’s average house value | 0.0025 |
| 51 | The number of allergies of the patient | 0.0024 |
| 52 | The number of families in the block group | 0.0024 |
| 53 | The number of distinct medication prescribers | 0.0024 |
| 54 | The change of weight in percentage | 0.0022 |
| 55 | The ellipsoid great circle distance between the patient’s home and the patient’s current PCP’s office | 0.0022 |
| 56 | The average SpO_2_ | 0.0022 |
| 57 | The area’s Hispanic population percentage | 0.0022 |
| 58 | Race = Asian | 0.0022 |
| 59 | The number of days since the last outpatient visit | 0.0020 |
| 60 | The total number of refills allowed in all of the medication orders | 0.0020 |
| 61 | The block group’s median monthly rent payment | 0.0019 |
| 62 | The number of laboratory tests | 0.0019 |
| 63 | The admit hour of the last ED visit | 0.0019 |
| 64 | Gender | 0.0018 |
| 65 | The combined population of each of the census blocks within the block group that qualifies as urban under the 2013 US census | 0.0018 |
| 66 | The percentage of single-parent households with dependents <18 years old in the block group | 0.0018 |
| 67 | Bronchiolitis | 0.0018 |
| 68 | The maximum temperature | 0.0017 |
| 69 | The block group’s median monthly mortgage payment | 0.0017 |
| 70 | The total length of all of the inpatient stays | 0.0017 |
| 71 | The number of asthmatic patients of the PCP | 0.0017 |
| 72 | Religion = Protestant | 0.0016 |
| 73 | The percentage of the civilian labor force 16 and older in the block group that is unemployed | 0.0016 |
| 74 | The percentage of households in the block group that are owner-occupied | 0.0015 |
| 75 | The number of civilian labor force 16 and older in the block group | 0.0015 |
| 76 | Whether the patient had any food allergy | 0.0015 |
| 77 | The PCP’s age | 0.0015 |
| 78 | The minimum SpO_2_ | 0.0015 |
| 79 | The estimated average number of people per household in the area | 0.0014 |
| 80 | Religion = Catholic | 0.0013 |
| 81 | The number of households in the block group | 0.0013 |
| 82 | The average systolic blood pressure | 0.0013 |
| 83 | The average temperature | 0.0012 |
| 84 | The area’s population size | 0.0012 |
| 85 | The total number of units of asthma reliever medications ordered that are neither systemic corticosteroids nor short-acting beta-2 agonists | 0.0011 |
| 86 | The number of chest X-ray exams | 0.0011 |
| 87 | The percentage of households in the block group without a motor vehicle | 0.0011 |
| 88 | The number of medication orders | 0.0011 |
| 89 | The area’s average household income | 0.0011 |
| 90 | The size of the population 25 and older in the block group | 0.0011 |
| 91 | The area’s average elevation | 0.0011 |
| 92 | The percentage of households in the block group with >1 person per room | 0.0011 |
| 93 | The primary payer’s insurance category at the patient’s last visit = other private insurance | 0.0010 |
| 94 | Smoking status = current smoker | 0.0010 |
| 95 | The number of no shows | 0.0010 |
| 96 | Whether the last inpatient stay was through the ED | 0.0009 |
| 97 | Whether the patient had any drug or material allergy | 0.0009 |
| 98 | The number of CPT procedure codes for pulmonary function tests | 0.0009 |
| 99 | The maximum respiratory rate | 0.0009 |
| 100 | The number of CPT/HCPCS procedure codes | 0.0009 |
| 101 | The acuity level of the last ED visit | 0.0008 |
| 102 | The number of days since having the last laboratory test | 0.0008 |
| 103 | The median home value in the block group | 0.0008 |
| 104 | The number of occupied households in the block group | 0.0008 |
| 105 | Religion = Christian | 0.0008 |
| 106 | The maximum heart rate | 0.0008 |
| 107 | The primary payer’s insurance category at the patient’s last visit = SelectHealth | 0.0007 |
| 108 | The percentage of population 25 and older in the block group with < 9 years of education | 0.0007 |
| 109 | The PCP’s primary specialty = family medicine | 0.0007 |
| 110 | The number of employed people 16 and older in the block group | 0.0007 |
| 111 | The area’s 2003 rural-urban continuum code | 0.0006 |
| 112 | The percentage of families in the block group that are below the federal poverty level | 0.0006 |
| 113 | The percentage of households in the block group without a phone | 0.0006 |
| 114 | The length of stay of the last ED visit | 0.0006 |
| 115 | Diabetes without chronic complication | 0.0006 |
| 116 | The number of days since the last inpatient stay | 0.0006 |
| 117 | The day of the week at the last ED visit’s admission time | 0.0006 |
| 118 | The maximum diastolic blood pressure | 0.0005 |
| 119 | The total number of refills allowed in all of the short-acting beta-2 agonist orders | 0.0005 |
| 120 | Religion = Baptist | 0.0005 |
| 121 | Smoking status = former smoker | 0.0005 |
| 122 | The number of cancelled appointments | 0.0005 |
| 123 | The estimated number of households in the area | 0.0004 |
| 124 | The PCP’s primary profession type = Doctor of Osteopathic Medicine | 0.0004 |
| 125 | Whether the patient had any environmental allergy | 0.0004 |
| 126 | The total number of refills allowed in all of the inhaled corticosteroid orders | 0.0004 |
| 127 | The maximum systolic blood pressure | 0.0004 |
| 128 | The total number of units of NSAIDs ordered | 0.0004 |
| 129 | Among the admission types of all of the visits of the patient, the one with the highest priority = urgent | 0.0004 |
| 130 | Chronic obstructive pulmonary disease | 0.0004 |
| 131 | Language = Spanish | 0.0004 |
| 132 | Obesity | 0.0004 |
| 133 | Marital status = single | 0.0004 |
| 134 | Upper respiratory tract infection | 0.0004 |
| 135 | The number of outpatient visits to the patient’s PCP | 0.0003 |
| 136 | The length of the last intensive care unit stay | 0.0003 |
| 137 | The number of visits that were referred | 0.0003 |
| 138 | The total number of refills allowed in all of the nasal steroid spray orders | 0.0002 |
| 139 | Breathing abnormality like dyspnea | 0.0002 |
| 140 | The block group’s rural/urban status | 0.0002 |
| 141 | The total number of antihistamines ordered | 0.0002 |
| 142 | The duration of chronic obstructive pulmonary disease | 0.0002 |

The 39 native machine learning classification algorithms in Weka: Bayes net, naive Bayes, naive Bayes multinomial, Gaussian process, linear regression, logistic regression, single-layer perceptron, stochastic gradient descent, support vector machine, simple linear regression, simple logistic regression, voted perceptron, *k*-nearest neighbor, *K*-star, decision table, RIPPER, M5 rules, 1-R, PART, 0-R, decision stump, C4.5 decision tree, logistic model tree, M5 tree, random forest, random tree, REP tree, locally weighted learning, AdaBoost M1, additive regression, attribute selected, bagging, classification via regression, LogitBoost, multiclass classifier, random committee, random subspace, voting, and stacking.

The hyper-parameter values of the XGBoost classification algorithm used in the final predictive model: alpha=1, Booster=gbtree, colsample_bytree=1, eta=0.3, eval_metric=auc, gamma=0, lambda=0, max.depth=4, min_child_weight=5, nrounds=100, objective=binary:logistic, scale_pos_weight=0.02, and subsample=1.

**References**

37. Populations studies center. Data sharing for demographic research knowledge base. 2017. https://dsdr-kb.psc.isr.umich.edu/answer/1102.

38. The Health Literacy Data Map homepage. 2019. http://healthliteracymap.unc.edu.

39. Singh GK. Area deprivation and widening inequalities in US mortality, 1969-1998. Am J Public Health 2003;93(7):1137-43. PMID:12835199
